# Impact of public hospital pricing reform on medical expenditure structure in Jiangsu, China: a synthetic control analysis

**DOI:** 10.1186/s12913-019-4357-x

**Published:** 2019-07-23

**Authors:** Xiao Zang, Mengran Zhang, Shihao Wei, Wenxi Tang, Shan Jiang

**Affiliations:** 1 Faculty of Health Sciences, Simon Fraser University, 8888 University Dr, Burnaby, BC V5A 1S6 Canada; 2 British Columbia Centre for Excellence in HIV/AIDS, 608-1081 Burrard St, Vancouver, BC V6Z 1Y6 Canada; 3 School of International Pharmaceutical Business, China Pharmaceutical University, No.639 Longmian Street, Nanjing, 211198 China; 4 School of Chinese Traditional Medicine, China Pharmaceutical University, No.639 Longmian Street, Nanjing, 211198 China; 5 School of Population and Public Health, University of British Columbia, 2206 East Mall, Vancouver, BC V6T1Z3 Canada

**Keywords:** Synthetic control, Health policy evaluation, Zero drug mark-up, Medical expenditure, Public hospital reform, China

## Abstract

**Background:**

The synthetic control method (SCM) is a useful tool in providing unbiased analysis on the policy effect in real-world health policy evaluations. Through controlling for a few confounding factors, we aim to apply SCM in analyzing the impact of the pricing reform on medical expenditure structure in Jiangsu Province, China.

**Methods:**

We constructed a synthetic control for Zhenjiang, a city where the reform was piloted in Jiangsu, by selecting weights on those potential control units to define a linear combination of the control outcomes to replicate the counterfactual as if the intervention is in absence. The policy effect was measured by the differences in the percentage of drug expenditure among average outpatient and inpatient care cost per visit in the post-policy period between Zhenjiang and its synthetic control. We also examined the significance of the estimated results by performing placebo tests, and cross-validated the results with a difference-in-differences analysis.

**Results:**

The medical pricing reform was found to be effective in reducing the drug expenditure proportions in both outpatient and inpatient care by an estimated mean level of 7.7 and 3.2% (or 16.3 and 9.2% relative decrease to their 2012 levels) respectively. This reform effect was estimated to be significant in the placebo tests and was further confirmed by a cross-validation.

**Conclusion:**

We conclude that the pricing reform in public hospitals has significantly reduced drug expenditure incurred in both outpatient and inpatient care. This study also highlights the applicability of SCM method as an effective tool for health policy evaluation using publicly available data in the context of Chinese healthcare system.

## Background

Real-world health policy evaluations often rely on evidence derived from natural experiments or observational studies where randomized control trials (RCTs) as the gold standard are ethically infeasible or practically unavailable [1]. Causal inferences are subject to potential confounding effects if non-RCT evidences are used; difficulties remain in choosing the proper comparison control groups and constructing the counterfactual that best resembles the characteristics of the case of interest [2].

Traditional time-series analysis lacks a control on the unobserved confounding factors, such as any policies/programs that occur after the target policy, and thus might incur bias in effect estimations [3]. Difference-in-differences (DiD) method is also commonly used to estimate the effect of a health policy, by contrasting the change in outcomes pre- and post-intervention, assuming the counterfactual treatment groups follow a parallel trend with the control groups [4, 5]. Nevertheless, such ‘parallel trend’ is sometimes criticized as an implausible assumption and thus might introduce bias to the results [6]. The synthetic control method (SCM) has emerged as an alternative to the DiD method that relaxes the parallel trend assumption. By allowing time-varying effect of unobserved factors, it is useful to evaluate the impact of a treatment or a pilot program of the treatment implemented in some regions or units [2, 6, 7]. This method has been widely applied in a variety of program evaluations worldwide [8–12]. Another major merit of SCM is that causal inference can be drawn from data collected at the aggregate level (public census and statistical yearbook), when individual-level data are unavailable or difficult to obtain.

China initiated a health system reform in 2009. The central component of the reform was the medical pricing reform, also known as the *zero-markup drug* policy, which removed the markups adding to the prices of any medications prescribed and sold in public hospitals. The drug mark-up policy could date back to 1955, when the policy allowed the public hospitals to add a 15% markup over the selling prices of any medications over their purchase prices [13]. The policy was issued to compensate the medical services in hospitals (e.g. diagnosis, nursing, surgery), which were underpriced due to the stable wage system back then. Unexpectedly, the policy incurred accumulating complains about over-prescription, especially in antibiotic products [14, 15], indicating the incentives of health providers’ services were distorted. The government therefore took actions to remove all the existing mark-ups and rectify the twisted pricing system of medications and medical services in the 2009 reform, aiming to control the proportion of drug in total medical expenditure and adjust the health providers’ behavioral incentives [16].

The reform of drug mark-up removal was initiated in 2010 nationwide and took on four phases before fully rolling out in September, 2017 [17]. Within each phase a number of cities were chosen to pilot the reform. Jiangsu initiated the reform in 2013, aiming to lower the proportion of drug expenditure while keeping the total medical expenditure per visit stable [18]. Zhenjiang was chosen as the pilot city in Jiangsu Province to implement the pricing reform on January 1, 2013, while all the other cities (11 cities in total except Suqian) in Jiangsu begun the reform on October 30, 2015.

Despite a few studies evaluating the reform [19–22], a formal unbiased analysis on the policy effects coupled with accurate construction for the counterfactual could identify the causality and ascertain the true values of the effects. In this study, we aim to apply the SCM in estimating the effect of the pilot reform in Zhenjiang by controlling confounding factors and weighting the control cities to construct a more suitable comparison group for Zhenjiang. Another purpose is to illustrate the usefulness of this method and its applicability in the evaluation of health policy in the context of Chinese healthcare system.

## Methods

### Synthetic control method

SCM uses longitudinal data to build a ‘synthesized control’ unit from weighted average of control units that best reproduces the characteristics of the treated unit, in both outcome variables and covariates prior to policy implementation, and as such construct the counterfactual outcomes for the treated by extrapolating the trajectories for the ‘synthesized control’ [6, 8]. In this study, we constructed a ‘synthetic control’ for Zhenjiang (the treatment unit), by selecting the weights of the remaining 12 potential control units in Jiangsu and as such defining a linear combination of the control outcomes to replicate the counterfactual as if the intervention was in absent. The control units (also defined as the donor pool) were composed of all the other cities in Jiangsu: Nanjing, Wuxi, Xuzhou, Changzhou, Suzhou, Nantong, Lianyungang, Huai’an, Yancheng, Yangzhou, Taizhou and Suqian. The weights were calculated by minimizing a distance matrix such that the synthetic control resembled the characteristics of Zhenjiang during the pre-policy period. The policy effect for each time point during post-policy period were then estimated by comparing the observed outcomes for Zhenjiang against the counterparts generated by the constructed ‘synthetic control’.

More specifically, the ‘synthetic control’ at time t (treatment-free), ${\hat{Y}}_{1t}^N$, was constructed by

$$ {\hat{Y}}_{1t}^N=\sum \limits_{j=2}^{J+1}{w}_j{Y}_{jt} $$

where the subscript *j* denotes the *j* th unit in the study, among which *j* = 1 stands for the treated unit and others for the control units;

*w*_*j*_ denotes the weight for unit j in constructing the ‘synthetic control’;

*Y*_*jt*_ denotes the observed outcome for unit j at time t;

$$ {w}_j\ge 0\kern0.5em and\kern0.5em \sum \limits_{j=2}^{J+1}{w}_j=1 $$

For a treatment initiated at time *T*_0_ upon unit 1, its effect at time t (*t* > *T*_0_) is estimated by:

$$ {\hat{\alpha}}_{1t}={Y}_{1t}-{\hat{Y}}_{1t}^N $$

The weight vector **W** is chosen to minimize the distance matrix:

$$ \sqrt{{\left({\boldsymbol{X}}_{\mathbf{1}}-{\boldsymbol{X}}_{\mathbf{0}}\boldsymbol{W}\right)}^{\prime}\boldsymbol{V}\left({\boldsymbol{X}}_{\mathbf{1}}-{\boldsymbol{X}}_{\mathbf{0}}\boldsymbol{W}\right)} $$

where, ***X***_**1**_ denotes a k ∗ 1 vector for the k covariates and pre-treatment outcomes for the treated unit;

***X***_**0**_ denotes a k ∗ J matrix for the corresponding covariates and pre-treatment outcomes for J control unites;

V denotes a k ∗ k positive definite and diagonal matrix that assigns weights according to the relative importance of the covariates and the pre-intervention outcomes. Such that:

$$ \sum \limits_{j=2}^{J+1}{w}_j{\boldsymbol{Z}}_{\boldsymbol{j}}={\boldsymbol{Z}}_{\mathbf{1}},\kern0.5em \sum \limits_{j=2}^{J+1}{w}_j{Y}_{jt}={Y}_{1t}\kern2.75em t=1,2,\dots {T}_0 $$

where, ***Z***_***j***_ denotes the vector of covariates for unit j.

In this study, the policy effect was measured by the changes in medical expenditure structure, defined by [1] the percentage change of drug expenditure relative to total outpatient cost per visit per person; [2] the percentage change of drug expenditure relative to total inpatient cost per visit per person. Since this reform was piloted in Zhenjiang on January 1, 2013 and was fully implemented across Jiangsu Province (except Suqian) in October 2015, we defined 2008–2012 as the pre-policy period and 2013–2015 as the post-policy period.

### Data source

We used publicly accessible data at city level. Data were obtained from multiple sources. For outcome variables, we used data from Jiangsu Health and Family Planning Statistical Yearbook between 2008 and 2016 [23]. For covariates of each city that were associated with the outcomes, we used data from Jiangsu Statistics Yearbook 2009–2017 [24], and Jiangsu Information and Development of the elderly population Report 2015 [25].

### Control variables

To construct the synthetic controls, we included variables that have been shown in previous studies to capture the confounding factors likely to affect the medical expenditure structure [19, 26]. We grouped these control variables in 2 categories: [1] the demographic and socioeconomic indictors, including population size, degree of urbanization (i.e. the proportion of urban population), proportion of the senior population (aged 60 and over), and GDP per capita (China Yuan, CNY); and [2] medical resource indicators, including life expectancy and governmental medical service operation expenses per capita (CNY). The operation expenses per capita was measured by the total expenses over the population size, as a proxy for the level of health insurance coverage. All the above covariate data were collected at the city level.

### Placebo tests

To estimate the statistical significance of the reform effect, we performed placebo experiments by iteratively re-assigning treatment status to each control unit and, for this ‘placebo-treated’ unit, re-estimating the treatment effect by applying the synthetic control method, then comparing the estimated treatment effect to the distribution of placebo effects [6]. We examined the proportion of placebo effects that were at least as extreme in absolute value as the estimated treatment effect for the unit of interest to assess whether the effect of medical reform in Zhenjiang was larger than that if the reform was randomly assigned to another city.

### Cross validation

To cross-validate the estimates of the reform effect derived from SCM, we performed an additional analysis using a DiD approach, which included the same set of outcome variable and control variables [4–6]. More specifically, the DiD model was constructed by:

$$ {Y}_{it}=\upgamma +{\beta}_1{Treat}_{it}+{\beta}_2{Post}_t+{\beta}_3{Treat}_{it}{Post}_t+b{X}_{it}+{\epsilon}_{it} $$

where *Y*_*it*_ represents the outcome variable for city *i* in year *t*, i.e. the proportion of drug expenditure in outpatient/inpatient care; *Treat*_*it*_ is a dummy variable representing treatment location (1 = Zhenjiang, 0 = other cities); *Post*_*t*_ is a dummy variable taking the value of 1 if year *t* is in the post-period and 0 otherwise; *X*_*it*_ denotes the covariates for the outcomes, which are the same set of control variables as in the SCM analysis; the terms *γ* represents the constant term, and *ϵ*_*it*_ represents the error term.

The treatment effect is the estimate of the coefficient *β*_3_. We applied separate DiD analyses in the outpatient and inpatient context.

## Results

**Fig. 1:**
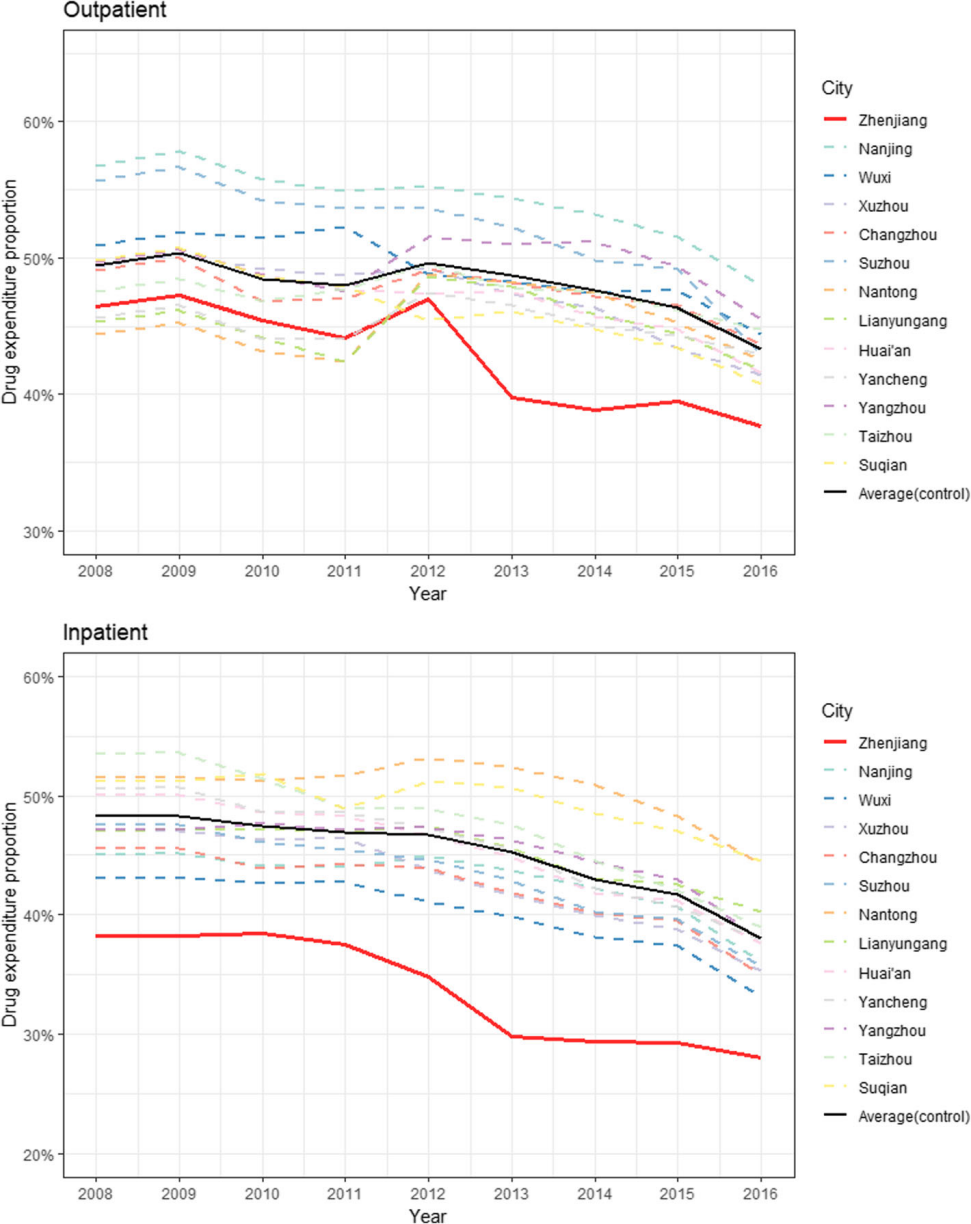
Longitudinal trend of the proportion of drug expenditure among total medical cost per visit. Legend: upper panel: outpatient care; lower panel: inpatient care

Figure 1 shows the longitudinal trend of the observed percentage of drug expenditure among total medical cost per visit in outpatient and inpatient care. Overall, we saw a decreasing trend in the drug expenditure proportion among both outpatient and inpatient care in most cities during the study period. As expected, the drug expenditure proportion witnessed a sharp decline in Zhenjiang in both outpatient and inpatient care in 2013 when the reform was piloted while the trends varied in other cities. Another turning point we noticed was 2016 where the drug expenditure proportion underwent a dramatic decline in all cities except Zhenjiang and Suqian, which was aligned with the fact that the same scale of reform that had been piloted in Zhenjiang was implemented to all the other cities except Suqian in 2015. However, we also noticed that the drug expenditure proportion among inpatient cost in Zhenjiang was significantly and constantly lower than other cities by an average of 10%, causing a synthetic control unable to be constructed from the donor pool, a methodological limitation noted by previous study [3]. Instead, we reduced this drug expenditure proportion by 10% for all cities in the donor pool in inpatient care to ensure comparability of baseline characteristics between the treated and control cities.

Table 1 compares the mean value of the outcome variable and covariates in Zhenjiang and the synthetic control, as well as the average of all cities in the donor pool during the pre-policy period. Overall, the synthetic Zhenjiang provided a better match on the characteristics in pre-policy period for outpatient care as compared to the average of other cities, particularly in reproducing the outcome variable. Similarly, in Table 2, with the adjustment made to the outcome variable, the synthetic Zhenjiang suggested a more suitable comparison group than the average of other cities for inpatient care.

**Table 1:** Means of city-level characteristics in pre-policy period: outpatient

Outpatient	Zhenjiang	Synthetic Zhenjiang	Other cities
Drug expense proportion: outpatient (%)	46.1	46.3	49.2
Population size (persons)	3,102,780	6,523,473	6,257,958
Degree of urbanization (%)	61.8	57.7	57.5
Elderly proportion (%)	23.7	24.8	21.1
GDP per capita (CNY)	65,808	56,248	51,899
Life expectancy (years)	80.22	80.86	79.16
Medical service expenses (CNY)	181.3	219.1	232.3

**Table 2:** Means of city-level characteristics in pre-policy period: inpatient

Inpatient	Zhenjiang	Synthetic Zhenjiang	Other cities
Drug expense proportion: inpatient (%)	37.5	37.5	37.5
Population size (persons)	3,102,780	5,093,626	6,257,958
Degree of urbanization (%)	61.8	59.6	57.5
Elderly proportion (%)	23.7	23.9	21.1
GDP per capita (CNY)	65,808	62,659	51,899
Life expectancy (years)	80.22	78.64	79.16
Medical service expenses (CNY)	181.3	188.7	232.3

Table 3 shows the weights of each city in the synthetic version of Zhenjiang. The composition differs for outpatient and inpatient synthetic Zhenjiang. The top three contributors to the synthetic Zhenjiang in outpatient care were Nantong (0.619), Wuxi (0.187) and Lianyungang (0.124), while the counterparts in inpatient care were Taizhou (0.412), Wuxi (0.299) and Yangzhou (0.250).

**Table 3:** City weights in the synthetic Zhenjiang

City	Weight (outpatient)	Weight (inpatient)
Nanjing	≈ 0	≈ 0
Wuxi	0.187	0.299
Xuzhou	≈ 0	≈ 0
Changzhou	0.069	0.038
Suzhou	≈ 0	≈ 0
Nantong	0.619	≈ 0
Lianyungang	0.124	≈ 0
Huai’an	≈ 0	≈ 0
Yancheng	≈ 0	≈ 0
Yangzhou	≈ 0	0.250
Taizhou	≈ 0	0.412
Suqian	≈ 0	≈ 0

**Fig. 2:**
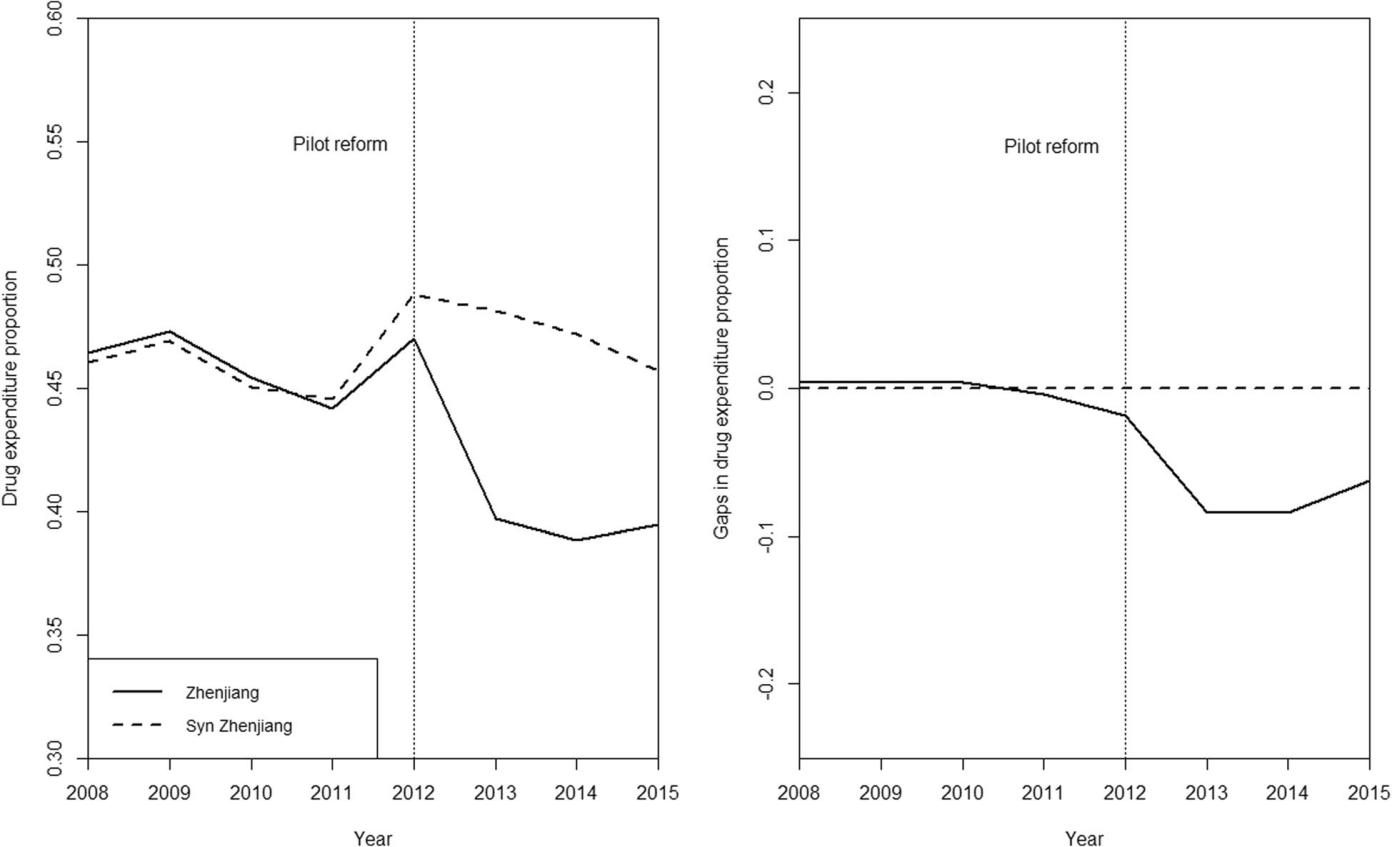
Graphical results of the synthetic control method for outpatient care. Legend: left panel: proportion of drug expenditure among average outpatient cost per visit: Zhenjiang VS. synthetic Zhenjiang; right panel: gap in drug expenditure proportion in outpatient care: Zhenjiang VS. Synthetic Zhenjiang

The left panel in Fig. 2 compares the proportion of drug expenditure among average outpatient cost per visit of Zhenjiang and its synthetic counterpart. The right panel demonstrates the intertemporal difference of this proportion between the observed and synthetic Zhenjiang during the study period. The drug expenditure proportion of the synthetic Zhenjiang remained stable from 2008 to 2015, while the actual data showed a decrease of 6.9% (from 46.4 to 39.5%). The mean reform effect was estimated to be 7.7% (absolute value in gap) during the post-policy period (2013–2015), which amounted to 16.3% of its 2012 level (47.0%).

**Fig. 3:**
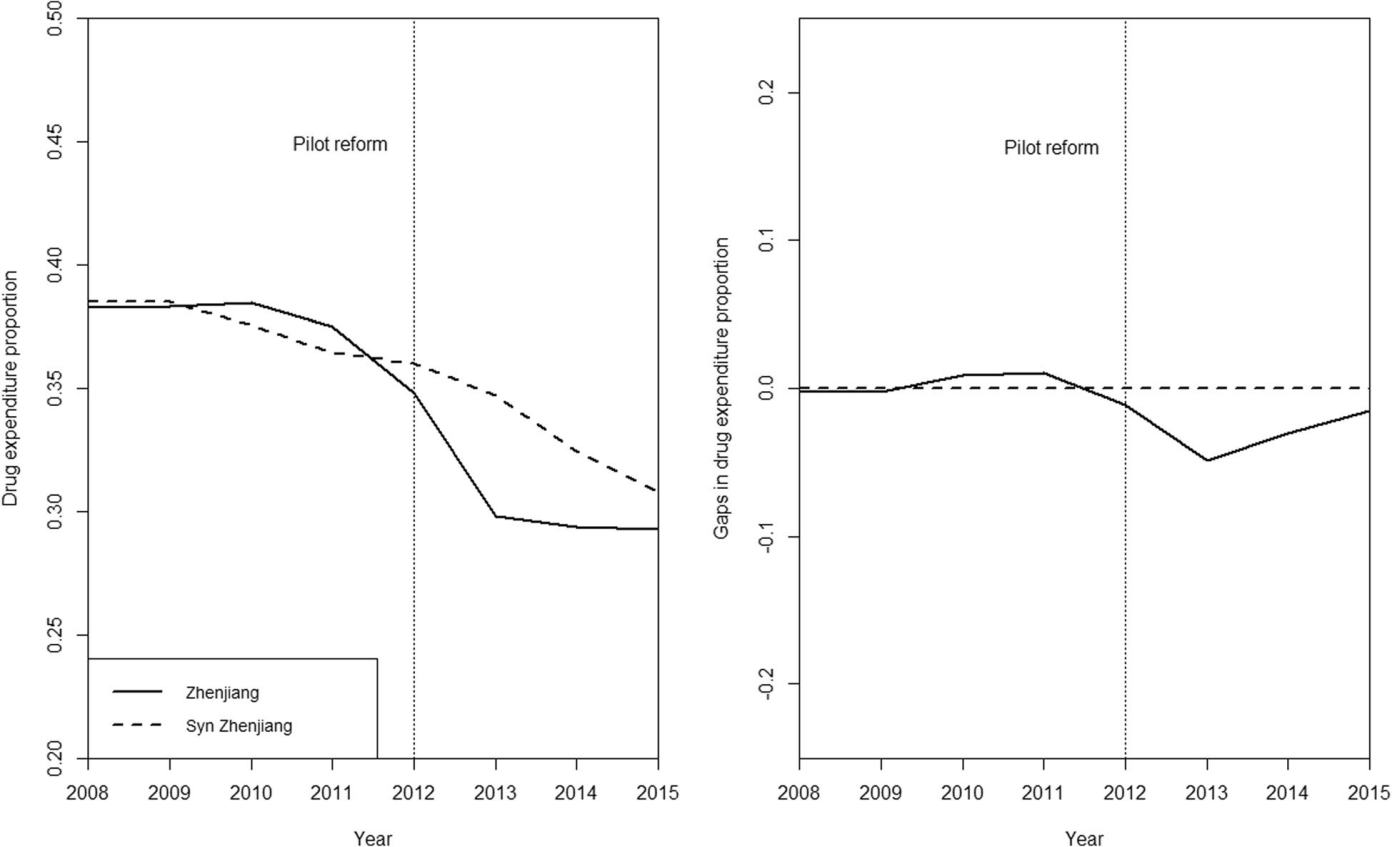
Graphical results of the synthetic control method for inpatient care. Legend: left panel: proportion of drug expenditure among average inpatient cost per visit: Zhenjiang VS. synthetic Zhenjiang; right panel: gap in drug expenditure proportion in inpatient care: Zhenjiang VS. Synthetic Zhenjiang

Figure 3 shows the comparison (left panel) and intertemporal difference (right panel) of drug expenditure proportion among average inpatient cost per visit between Zhenjiang and the synthetic Zhenjiang. The proportion dropped in both groups, whereas the decline was greater in the observed Zhenjiang (9.0%, from 38.3 to 29.3% between 2008 and 2015). The results suggested an estimated mean reform effect of 3.2% in absolute value (2013–2015) or 9.2% decrease proportional to its 2012 level (34.8%), suggesting that the reform might pose a larger impact on outpatient care medical expenditure than on inpatient care.

**Fig. 4:**
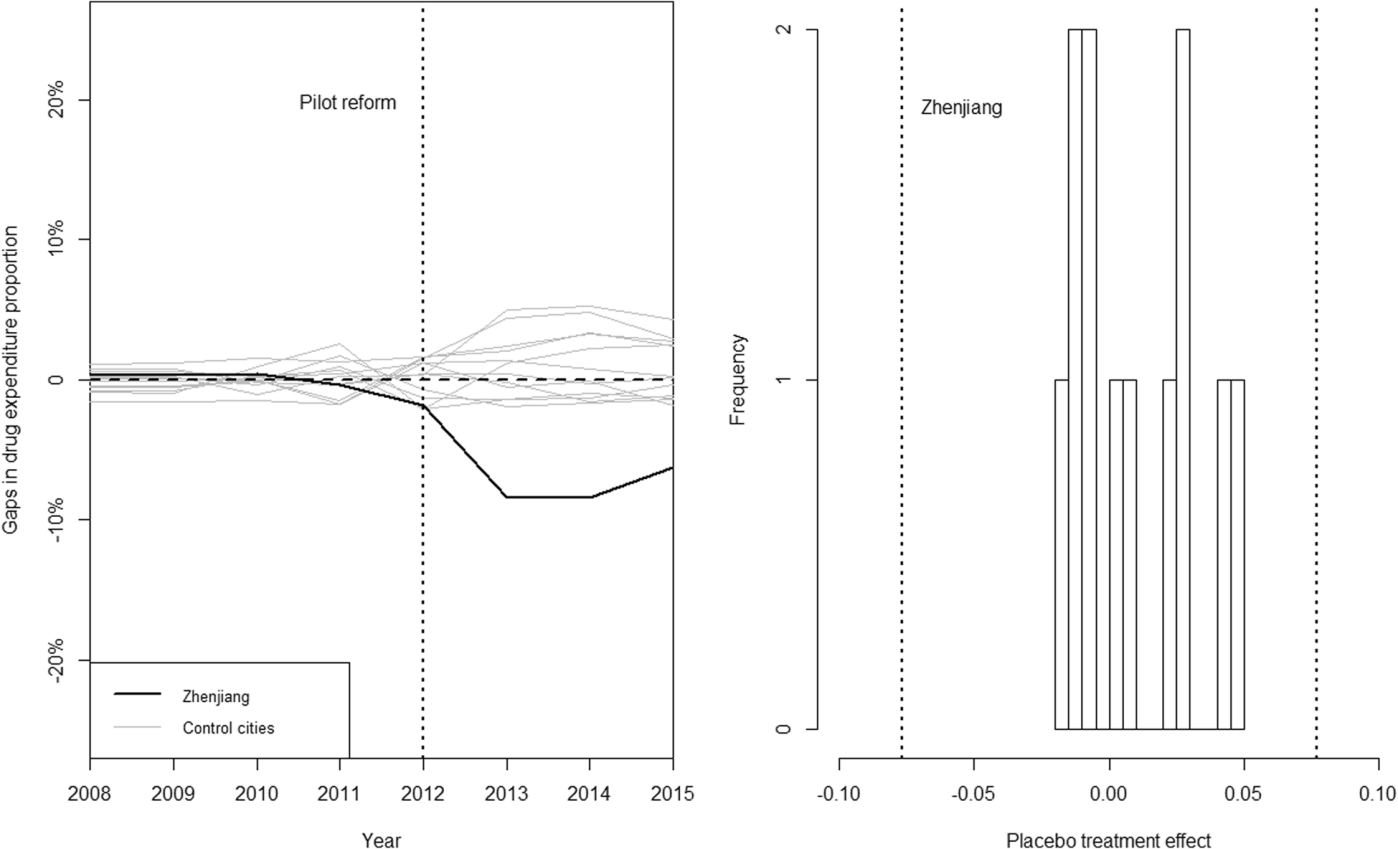
Graphical results of the placebo tests for outpatient care. Legend: left panel: placebo effects: Zhenjiang VS. control cities; right panel: histogram for placebo effects. The histogram summarized the distribution of estimated placebo effects for control cities. The dashed lines indicate the estimated mean reform effect for Zhenjiang and its opposite value

**Fig. 5:**
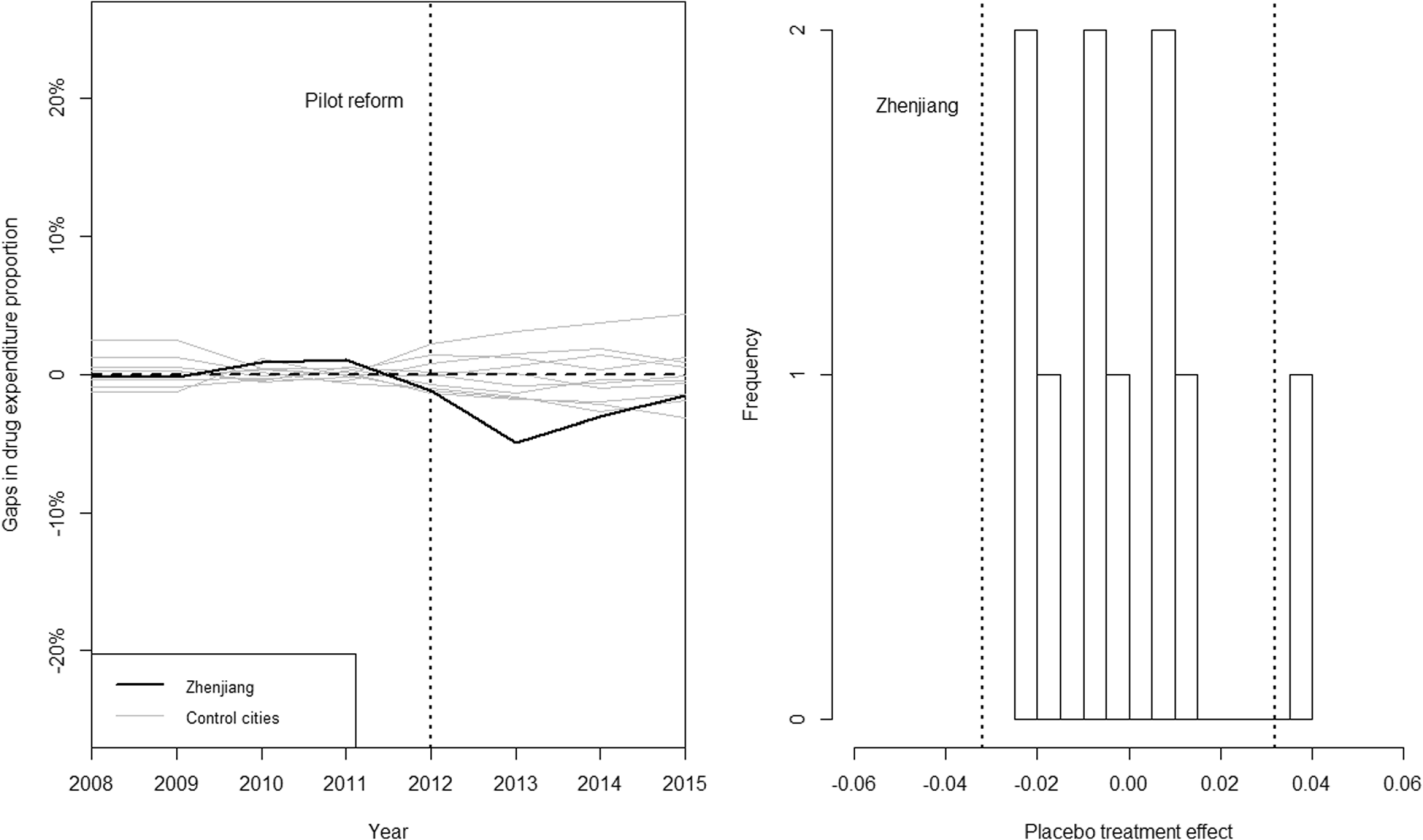
Graphical results of the placebo tests for Inpatient care. Legend: left panel: placebo effects: Zhenjiang VS. control cities; right panel: histogram for placebo effects. The histogram summarized the distribution of estimated placebo effects for control cities. The dashed lines indicate the estimated mean reform effect for Zhenjiang and its opposite value

None of the placebo effects were found to be as great as the estimated effect in Zhenjiang in outpatient outcomes (Fig. 4, left panel), while there was only one city, Suqian, whose placebo effect was greater that Zhenjiang in inpatient scenario (Fig. 5, left panel), indicating a significant policy effect of the medical reform. To note that, in conformity with the standard practice of Abadie et al. [7], we excluded cities whose pre-policy mean squared prediction error (MSPE) of the drug expenditure proportion that were greater than 5 times the MSPE that we found in Zhenjiang in inpatient scenario in the placebo test analysis. We interpreted the seemingly large placebo effects in these excluded cities as a result of a lack of fit, rather than of the effect of the reform. As a result, placebo test results for Wuxi and Nantong were excluded in our analysis for inpatient care. We also found that the trajectories of the placebo outcomes were distributed symmetrically around zero for both outpatient and inpatient care (Fig. 4, right panel and Fig. 5, right panel), suggesting that the synthetic control method would be expected to estimate a zero effect when the true effect was zero.

Our cross-validation using a DiD method also confirmed our estimates for the impact of the pricing reform, showing an estimated 4.9% (95% confidence interval: 1.2–8.8%, *p*-value: 0.010) and 3.2% (95% confidence interval: 0.094–6.3%, p-value: 0.043) reduction in the proportion of drug expenditure in outpatient and inpatient care, respectively.

## Discussion

This synthetic control analysis has shown significant impact of the medical pricing reform on medical expenditure structure in Zhenjiang in reducing the proportion of drug expenditure among total medical cost per visit in both outpatient and inpatient care. The results are consistent with previous studies. For instance, in 2014, Wang et al. forecasted that there would be a decrease in the proportion of drug expenditure over the per-visit in-patient care expenditure from 56.8 to 47.8% using data from one pilot district of Nanjing [27]. In 2017, Hu et al. analyzed the per-visit drug expenditure proportion in out-patient care in 5 cities in Jiangsu, and found consistent trend of decrease in this proportion: Nanjing (60.9 to 55.7%), Xuzhou (62.4 to 57.0%), Suzhou (54.2 to 48.5%), Nantong (50.5 to 45.5%) and Huai’an (50.0 to 43.6%), ranging from 5.0 to 5.7% [28]. In 2015, Dai et al. stated that the drug expenditure proportion of the per-visit expenditure in out-patient and in-patient care was estimated to drop by 6.0% (46.3 to 40.3%) and 4.8% (34.5 to 29.7%) from 2012 to 2013, respectively, in Zhenjiang [29]. It is worth noting that the studies mentioned above might have limitations of not controlling for individual trends or confounders. On the contrary, our study explicitly accounted for socioeconomic confounders to construct a more suitable counterfactual for comparison, and we estimated the effects to be 7.7% (outpatient) and 3.2% (inpatient), indicating that previous studies presented potentially biased effects of the pricing reform.

However, this study is not without limitations. First, in some control cities, the reform was implemented at scattered levels (i.e., in some regional hospitals) during the pre-policy period (2012–2015), which would inevitably reduce the estimated impact. However, we found significant results from such conservative estimation, further reinforcing the reform effect. Second, as shown in Fig. 1 (lower panel), Zhenjiang displayed strong uniqueness in inpatient care expenditure structure, due to which a synthetic counterfactual was unable to construct from the donor pool [3]. We instead reduced the proportion of inpatient drug expenditure for control cities by a fixed number to enable comparability between the treatment and control groups. However, bias could be introduced by the adjustment with respect to the magnitude of the estimated effect. To address this potential bias, we conducted an additional cross-validation with a DiD approach that confirmed the estimated effect size.

This study contributes to literature in the following ways. First, the evaluation of health policies and reforms in China has long been limited by data unavailability. The access of individual-level data, as the gold standard of data source, was rather restricted by health authorities and hospitals. The synthetic control analysis of this study in the context of Chinese healthcare system provides an example of how to utilize publicly available population-level data and reduce the reliance on individual level data on to evaluate the effect of health policies and reforms. Second, some previous evaluation studies had less control over confounders, which might over- or under-estimate the impacts of certain policies. This study provides an analytic framework to account for a series of covariates to exclude potential confounding effects. Third, our conservative results suggest that the impact of pricing reform in Jiangsu was potentially underestimated, which might arouse the needs to further examine the impacts of similar reforms in other Chinese provinces. Last, we hope this study could provide meaningful implications for the applicability of the synthetic control analysis method in future policy evaluation and decision-making in China.

## Conclusions

We demonstrate the applicability of the SCM by studying the effects of Jiangsu’s medical pricing reform on medical expenditure structure through a case study in the city of Zhenjiang where the reform was piloted. This study suggests that the medical pricing reform in urban public hospitals has significantly reduced drug expenditure incurred in both outpatient and inpatient care. While our results are generally consistent with other studies, this SCM analysis contributes to the literature by rigorously selecting comparison units, constructing counterfactual scenarios and controlling for confounding effects. This study also highlights the value of population-based approach that can be constructed from publicly available census data on the evaluation of health policy and reform.

## Data Availability

The datasets used and/or analyzed during the current study are available from the corresponding author on reasonable request.

## Abbreviations

DiD: Difference in difference
GDP: Gross domestic product
MSPE: Mean squared prediction error
RCT: Randomized controlled trial
SCM: Synthetic control method
